# Clinical, virological and epidemiological characterization of an outbreak of Testudinid Herpesvirus 3 in a chelonian captive breeding facility: Lessons learned and first evidence of TeHV3 vertical transmission

**DOI:** 10.1371/journal.pone.0197169

**Published:** 2018-05-10

**Authors:** Maria Luisa Marenzoni, Lorenzo Santoni, Andrea Felici, Carmen Maresca, Valentina Stefanetti, Monica Sforna, Maria Pia Franciosini, Patrizia Casagrande Proietti, Francesco Carlo Origgi

**Affiliations:** 1 Department of Veterinary Medicine, University of Perugia, Perugia, Italy; 2 Tartoombria, Foligno, Perugia, Italy; 3 Istituto Zooprofilattico Sperimentale of Umbria and Marche, Perugia, Italy; 4 Institute of Veterinary Bacteriology, Centre for Fish and Wildlife Health, Vetsuisse Faculty, University of Bern, Bern, Switzerland; University of California Riverside, UNITED STATES

## Abstract

Testudinid herpesviruses (TeHVs) have a worldwide distribution among tortoises. However, information such as risk factors promoting the occurrence or the recrudescence of the associated disease and the mid-term sequelae of an outbreak comprising the extent and dynamic of the viral shedding have been only minimally investigated. Critical management information is also lacking or anecdotal. Furthermore, major aspects of the viral pathogenesis including the likelihood of vertical transmission of the virus are virtually unknown. The present study describes the occurrence and the management of an outbreak of *Testudinid herpesvirus* genotype 3 (TeHV3) in a large, private collection of chelonians. Clinical, pathological, molecular and serological characterization of the outbreak were carried out. Seventy-five percent of the infected tortoises died. Complementation of molecular and serological testing was a critical point for successful management implementations. A case-control study was performed to analyze possible risk factors associated with the infection. Furthermore, a subgroup of six asymptomatic infected tortoises was monitored for two consecutive seasons after the outbreak: all the infected tortoises were determined to be intermittent shedders, except one, which was a persistent shedder. Post-hibernation was associated with the highest number of shedders. Finally, evidence of the most likely vertical transmission of the virus was obtained for the first time.

## Introduction

Herpesviruses are among the most important and globally distributed viral pathogens in chelonians [1–3]. However, only partial knowledge regarding the etiologic agents, associated epidemiology and pathogenesis is currently available.

*Testudinid herpesviruses* (TeHV, -1, -2, -3, and -4) comprise four distinct genotypes infecting the members of the subfamily *Testudinidae* (tortoises). Of them, TeHV3 has recently been proposed as member of the genus *Scutavirus*, in the subfamily *Alphaherpesvirinae* [4,5]. Collectively, these four herpesviruses are able to virtually infect any tortoise [1,3,6–8]. TeHV1 and 3 have been detected in Europe, Asia and USA, TeHV2 in the USA, and TeHV4 in tortoises imported from Africa [3,8]. TeHV3 has been reported as more virulent than TeHV1, although no specific transmission studies comparing the two genotypes have been carried out so far [2,3].

A number of TeHV-3 outbreaks have been investigated. However, several aspects of the infection are still poorly understood [3, 9]. Clinical signs include conjunctivitis, nasal and/or oral discharge, diphtheronecrotic stomatitis, glossitis, and pharyngitis. Neurological signs consisting of lethargy, circling, paralysis and incoordination have been reported [2,3,10]. There is indirect evidence for the virus’ establishing lifelong latency in the nervous system upon primary infection with consequent persistence in the infected host population [3,11]. Reactivation of the latent virus can occur with or without clinical signs [11].

In order to attempt to fill these gaps we investigated and characterized the events that occurred during an outbreak of TeHV3 in a large non-commercial breeding facility. Clinical, serological, virological, and epidemiological data obtained throughout the outbreak are reported here along with the measures undertaken to contain the disease. Possible risk factors were investigated. Finally, the first evidence of the most likely TeHV3 vertical transmission is provided in this article.

## Materials and methods

### Ethics statements

All the data presented in this study were collected during routine diagnostic procedures and consequently no ethical permission was required. The owner of the tortoises gave full permission to use the data collected from the animals involved in this study.

### Animals, housing and breeding facility

At the time of the outbreak, in April 2013, the breeding facility consisted of 314 chelonians, including 256 *T*. *hermanni*, 7 *T*. *graeca*, 4 *T*. *horsfieldii*, 3 *T*. *marginata*, 41 turtles (16 *Trachemys* spp., 14 *Emys orbicularys*, 5 *Graptemys* spp., 4 *Pseudemys* spp., 2 *Cuora amboinensis*) and 3 *Terrapene carolina mayor*. The original founders of the breeding colony consisted of 7 *T*. *hermanni*, of unknown origin, raised since the 1970s, kept as pets in private household backyards and notified to the Italian authorities (Corpo Forestale dello Stato) according to the regulations derived from the Convention on International Trade in Endangered Species of Wild Fauna and Flora (CITES) in 1992–1995. The number of tortoises in the collection increased thanks to new births and some occasional donations over the years. The animals were identified by microchip in accordance with national Italian laws (CITES legislation; Regulation EC 338/97, with modifications; Italian Law no. 150/1982, with modifications) and photographed during the first year of life. Non-toxic enamel paint was used to mark the shell of the hatchlings before they could be identified by microchip. A one year-long quarantine was carried out for each new arrival. Following quarantine, these animals were placed into a fenced enclosure with young individuals that served as sentinels for possible infections. The access to the breeding colony was limited to the owner and his family only. No history of mortality or obvious disease were reported in the past and a low mortality was recorded for yearlings only and was considered within normal limits (<1%). The breeding colony has not commercial purpose.

All animals were housed outdoors. Tortoises were divided into small groups according to species and age, and housed in 20 independent fenced enclosures, 13 of which were in a restricted area and separated by a 40 cm high plasterboard wall (S1 Fig). Turtles lived in fenced enclosures that also contained small artificial ponds (S1 Fig, enclosures nos. 9 and 10). Some of the oldest tortoises lived in the central enclosures (S1 Fig, enclosures nos. 1–4) and others in the outer ones (S1 Fig, enclosures nos. 7, 8, 11). Most of the younger animals were housed in enclosures surrounding the center of the facility, serving as sentinels (S1 Fig, enclosures nos. 5, 6, 12, 13). A net covered the enclosures to avoid access to predatory birds. The movements outside each enclosure were limited to adult animals for reproduction purposes only. The enclosures were regularly monitored during the year by a camera (Brinno TimeLapse Camera-GardenWatchCam GWC, Taiwan) to check for mating. The tortoises were fed with locally raised vegetables. The owner wore gloves and overshoes when managing animals and handling food, water, and equipment.

Eggs laid underground were regularly removed and incubated at 24–33°C with 70–90% humidity for 60–90 days. However, some eggs remained undetected underground, and consequently, they hatched within the enclosure. All newborn tortoises were kept separated from adult and young animals, so the hatchlings and the youngest tortoises shared the fences only with animals of the same age.

### Epidemiological investigation and definitions

A descriptive epidemiological study was carried out on the TeHV outbreak, which occurred in April-August 2013 and monitored until December 2014. The peak of the outbreak was further divided into an initial phase, until 13 April 2013, and a second phase, after 13 April 2013, based on the procedures undertaken by the owner on that date. The overall interval of time between April 2013 and December 2014 is defined in the study as the “outbreak period”.

The period of seasonal activity of the tortoises was divided into four periods, according to Cheylan [12] and Hout-Daubremount and Grenot [13], and more specifically into “post-hibernation” from March to April; “spring” from May to June; “summer” from July to August; and “pre-hibernation” from September to October, respectively.

During the period of observation, the following data were collected for each animal: animal ID, age, gender, enclosure, date of the onset of clinical signs, type of clinical signs, outcome, treatment, and laboratory test results.

Suspected cases were defined as animals with clinical signs (oral and/or nasal discharge; oral plaques) during the outbreak period. Confirmed cases were defined as cases with clinical signs and detection of TeHV DNA by polymerase chain reaction (PCR). Infected animals were defined as animals with a positive TeHV PCR result from oral swab (OSW) or tissues, independently from the presence of clinical signs. Exposed animal were defined as animals that had contact with confirmed cases or infected animals, as mentioned above. Considering the viral host-range [3], only the members of the *Testudinidae* family were considered the population at risk of infection (n = 270) and investigated in this study.

The following epidemiological parameters concerning the outbreak were defined in accordance to Thrusfield, 2005 [14]: cumulative incidence (the proportion of tortoises positive by PCR for TeHV on OSW or tissues in the population at risk during the outbreak period); incidence rate (the number of new infected cases that occurred in the population at risk during the first year of observation divided by the sum, over all individuals, of the length of time at risk of developing the infection and expressed as animal-time unit, month in this case); morbidity (the proportion of confirmed cases in the population at risk during the outbreak); mortality (the proportion of dead tortoises and PCR positive at the time of the death in the population at risk); case-fatality (the proportion of the PCR positive dead tortoises out of the infected tortoises during the outbreak).

### Post-mortem examination

When possible, the dead tortoises were subjected to necropsy and representative tissues were fixed in 10% neutral buffered formalin, routinely processed, embedded in paraffin, sectioned 5 μm thick and stained with haematoxylin and eosin for histopathological examination, according to standard protocols.

### PCR and sequencing

OSWs and, when possible in the case of dead animals, tissues (a pool of liver, spleen, and lung) were collected from the animals. OSWs were obtained using sterile cotton swabs, kept in tubes with 0.5 mL of phosphate buffered saline (PBS), and submitted for detection of TeHV DNA by PCR. Specimens were processed individually or in very little groups on the date of the admission in the laboratory.

Two hundred μl of OSW transport buffer (PBS) or 20 mg of tissues of dead tortoises were used for DNA extraction using a commercial kit (DNeasy Tissue kit, Qiagen, Italy). The concentration and purity of the extracted DNA was quantified using a NanoDrop® spectrophotometer (NanoDrop 2000, Thermo Fisher Scientific, Italy).

Initially, a nested, consensus, pan-herpesvirus PCR, targeting the partial sequence of the DNA polymerase gene, was used as a screening PCR considering the wide range of possible herpesviruses existing in chelonians [15]. Following the identification of the infecting genotype as TeHV3 by sequencing, a confirmatory panel of TeHV3-(genotypic)-specific PCRs (DNA helicase and ribonucletide reductase large subunit, UL39) was used [6,11]. Finally, a semi-nested PCR for the detection of TeHV3 was developed from the protocol targeting the UL39 gene [11] to increase its sensitivity. Details of the PCR protocols are provided in the S1 Table.

An aliquot of 10 μl of OSW DNA or 100 ng of DNA from tissues were tested in duplicate in a PCR (Microtech, Italy). Twenty-five μL of reaction mixture contained 10x buffer, 3 mM MgCl_2_, 200 μM each deoxyribonucleotide triphosphate, 1 μM each primer (Sigma-Genosys, Italy), 0.5 U Taq DNA polymerase (Microtech, Italy), and DNA template as described above. Either 5 or 1 μL of DNA of the first run was used for the nested protocol [15] or the semi-nested PCR (the new protocol of the present study), respectively. Cycling conditions are reported in the S1 Table. In each set of reactions, a positive control (a positive sample confirmed by sequencing) and a negative control (OSW of a negative healthy tortoise previously tested), as well as a negative reaction mix control (containing the reagents and water instead of DNA), were included. Serial 10- and 2-fold dilutions of the TeHVs–positive sequenced samples were carried out to determine the difference in sensitivity of the conventional and nested/semi-nested PCR protocols.

The PCR products of the expected size were purified with an extraction kit (Qiaquick PCR purification kit, Qiagen, Milan, Italy) and directly sequenced on both strands respectively with the same primers previously described, or with the primer TGVseq (5'-CATCTGATGTAACTCGGTGTA-3') and IYGseq (5'-GACAAACACAGAGTCCGT-3') in the case of the consensus pan-herpesvirus PCR [15], using a DNA analyzer (ABI 3730, Applied Biosystems) capillary sequencer (Primm srl and Bio-Fab Research srl, Italy). The sequences were assembled and aligned using BioEdit software and sequence similarities were assessed by comparison with the sequences deposited in GenBank using the BLAST software (https://blast.ncbi.nlm.nih.gov/Blast.cgi). Furthermore, all the sequenced amplicons (n = 4) corresponding to the partial sequence of the UL39 gene obtained by infected individuals were aligned and compared with each other using the MAFFT software (European Bioinformatics Institute, EMBL, https://www.ebi.ac.uk/Tools/msa/mafft/).

Finally, the identification of TeHV3 genogroup of the detected TeHV3 strain was determined by partial sequencing of the glycoprotein B (gB) gene and identification of the associated molecular signature as previously described [5].

### Enzyme-linked immunosorbent assay (ELISA)

After four months from the onset of the outbreak, a serological investigation was performed to confirm or rule out the exposure of the surviving tortoises to TeHV3 and to evaluate the possible persistence of the virus in the collection. This 4-month period was twice as long as recommended to test suspected animals and was selected in order to avoid any possible false negative results, especially in the presence of potential slow seroconverting individuals [3,16].

Blood samples were collected from the cervical dorsal sinus into sterile, plain Vacutainer tubes (Becton Dickinson, Milan, Italy). Sera were obtained by centrifugation at 6000 x*g* for 1 min and tested by ELISA, which was performed according to an established protocol [16]. Briefly, ELISA plates (Nunc Maxisorp, Germany) were coated with whole TeHV3 viral proteins derived from Terrapene heart cells (TH-1, CCL-50, American Type Culture Collection) infected with the strain US1976/98 and prepared at a concentration of 5 μg/ml in 0.01 M sodium phosphate buffer (pH 7.2), containing 0.15 M NaCl and 0.02% NaN_3_ (PBS-A), and incubated overnight at 4°C. Following, the antigen was discarded and 300 μl of blocking buffer (5% nonfat dry milk in PBS-A) was added to each well and left in the plate for 1 h at room temperature. Tortoise sera were diluted in blocking buffer at a final dilution of 1/25 and then added to the wells and incubated for 1 h at room temperature. Following three washes with PBS-A containing 0.05% Tween 20 (PBS-T), a mouse anti-tortoise immunoglobulin (HL1546), diluted at a final concentration of 1 μg/ml in PBS-A, was added to the wells and incubated for 1 h at room temperature. Following three washes in PBS-T, a secondary antibody goat anti/mouse alkaline phosphatase (AP)-labeled was added (1/1000 dilution in PBS-A) and incubated for 1 h at room temperature. Following three more washes with PBS-T, 100 μl of the AP-substrate (*p*-nitrophenyl phosphate disodium 1 mg/ml, prepared in 0.01 M sodium bicarbonate buffer [pH 9.6], containing 2 mM MgCl_2_; Sigma-Aldrich, Germany) was added and incubated for 1 h at room temperature. This test was validated for Greek and Hermann’s tortoises [11,16]. The sera used as positive and negative controls were those obtained during the original transmission study carried out on Greek tortoises infected with TeHV3 [11]. The optical density (OD) reading was recorded at 405 nm wave-length with a spectrophotometer (Genesys 20 Spectrophotometer, Thermo Scientific, Milan, Italy). The cutoff for positive samples was defined as the mean (A_405_) plus three times the standard deviation calculated on the sera samples collected from the naïve uninfected tortoises prior of the experimental infection carried out in a previous investigation [11,16]. The final cutoff value was determined to be 0.48 (OD A_405_). Doubtful sera samples in the current investigation were interpreted as those, which had one of the replicates over OD A_405_ 0.48 but not the other one, and for those for which this ambiguity could not be solved even after the repetition of the test.

### Risk factor analysis

A case-control study was performed to investigate possible risk factors relevant for TeHV3 infection occurrence. The variables considered included: tortoise species (*T*. *hermanni vs T*. non-*hermanni*, without subspecies level), gender, age, and location of the tortoises in the enclosures. In this study design, cases were considered the infected animals, independently of the presence of clinical signs. Three controls for each case were randomly selected from healthy resident tortoises housed in enclosures adjacent to those where the outbreak began. Animals housed in enclosures far from those affected by the outbreak were excluded from the present analysis because they were considered less likely to be infected. Attack rates, specific for each considered variable, and corresponding *p-*values obtained using chi-squared test or Fisher exact test, as appropriate, were calculated. Odds Ratios (OR) were calculated as crude measure of the association between each single risk factor and outcome (presence of infection). The assessment of age, gender and species as confounding factors was estimated by the Mantel-Haenszel method using Episheet (http://krothman.hostbyet2.com/episheet.xls).

The relationship between temperature and infection was investigated using a time-stratified case-crossover design. The detailed description of methods and results of this part is reported in the S1 Appendix.

### Retrospective investigation

OSW DNA of 38 adult tortoises housed in the facility and collected prior the outbreak, during post-hibernation in April 2011, and then stored at -20°C, was retrospectively investigated for the presence of TeHV genomic DNA.

## Results

### Description of the outbreak

The index case (tortoise no. 1, Table 1) was a male *T*. *hermanni hermanni*, aged over 30 years, with nasal discharge, that died on April 11, 2013. Initially, this was considered a sporadic death and consequently not further investigated. The next day, other animals became ill and died within several days. The list of events that occurred during the outbreak are reported in Table 1. Neurologic disease, consisting of hind limb paresis (S1 Video), was present in one animal (no. 13).

**Table 1:** Features of exposed tortoises during the TeHV3 outbreak.

ID tortoise	species	sex	age (years)	location 2012 (ID enclosure)	location 2013, initial phase (ID enclosure)	location 2013, second phase (ID enclosure)	date of onset of clinical signs	list of clinical signs	date of death	herpes-viruses PCR result	serological result	antiviral treatment^
1	*Testudo hermanni hermanni*	M	>30	1	3	0	April 07, 2013	ND	April 11, 2013	+	nc	-
2	*Testudo hermanni hermanni*	M	>30	1	2	0	April 12, 2013	OD	April 20, 2013	+	nc	-
3	*Testudo hermanni boetgerii*	M	10	1	4	0	April 12, 2013	OD	April 14, 2013	+	nc	-
4	*Testudo hermanni hermanni*	M	>30	1	1	0	April 12, 2013	OD	April 22, 2013	+	nc	-
5	*Testudo hermanni hermanni*	M	10	1	1	0	April 13, 2013	OD; OP	April 14, 2013	+	nc	-
6	*Testudo hermanni boetgerii*	M	10	1	4	4	April 14, 2013	OD	April 16, 2013	+	nc	-
7	*Testudo hermanni boetgerii*	M	10	1	4	4	April 14, 2013	OD	April 17, 2013	+	nc	-
8	*Testudo hermanni boetgerii*	M	>30	1	4	4	April 20, 2013	OD	April 25, 2013	+	nc	-
9	*Testudo hermanni hermanni*	M	9	1	4	4	April 20, 2013	OD	April 26, 2013	+	nc	-
10	*Testudo hermanni hermanni*	M	9	4	4	4	April 20, 2013	OD; ND	June 17, 2013	+	nc	-
11	*Testudo hermanni hermanni*	F	>30	2	2	3	April 28, 2013; May 19, 2013§	OD; ND§	May 21, 2013	+	nc	-
12	*Testudo hermanni hermanni*	F	>30	2	1	2	May 23, 2013	OD; OP	June 22, 2013	+	nc	+
13	*Testudo hermanni hermanni*	F	>30	2	1	2	August, 2013	OD; hind limb paresis#	March 17, 2014	+	nc	+
14	*Testudo graeca ibera*	F	>30	2	1	1	-	No	-	+ IS	nc	+
15	*Testudo hermanni hermanni*	F	>30	2	1	2	-	No	July, 2013	+	nc	+
16	*Testudo hermanni hermanni*	F	10	2	3	2	-	No	-	+ IS	nc	-
17	*Testudo hermanni hermanni*	F	>30	2	3	2	March, 2014†	ND†	March, 2014	+ IS	nc	-
18	*Testudo marginata*	M	15	1	2	4	March 17, 2014†	ND†	August, 2014*	+* IS	+	-
19	*Testudo graeca ibera*	M	16	4	4	4	-	No	-	+ PS	+	-
20	*Testudo hermanni hermanni*	F	>30	2	1	2	-	No	-	+ IS	+	-
21	*Testudo hermanni hermanni*	F	>30	2	2	2	-	No	-	-	-	-
22	*Testudo hermanni hermanni*	F	>30	2	2	2	-	No	-	-	-	-

M: male; F: female

ND: nasal discharge; OD: oral discharge; OP: oral plaques.

IS, intermittent shedder: the tortoise was alternatively positive or negative during the period of monitoring that lasted until December 2014 or until their death (whichever came first).

PS, persistent shedder: the tortoise was consistently positive during the period of monitoring that lasted until December 2014.nc = not conducted. These tortoises died before that the serological tests could be carried out.

^ Antiviral treatment consisted of acyclovir 80 mg/kg/day *per os* for 30 days.

†: Asymptomatic in the first year of the outbreak and with mild clinical signs the following year.

§: It had recurrent clinical signs

# hindlimb paresis (S1 Video) lasted all year long, even if with partial improvement. At the beginning of the occurrence of the paresis, the tortoise was able to lay 3 eggs. This tortoise partially recovered from the neurological signs, but died during post-hibernation the following year, after having had oral discharge. It was repeatedly positive for TeHV DNA (from OSW) during all the period until its death.

*The PCR was positive during the monitoring but not at the time of the death of the tortoise.

Oral discharge, characterized by the presence of dense mucus, was the main clinical sign, reported in 12 out of the 15 confirmed cases (80%). Nasal discharge (5/15, 33.3%), oral diphtheronecrotic plaques (2/15, 13.3%), neurologic signs (tortoise no. 13) were also present. The majority of the cases (9/15, 60%) died within 10 days from the onset of clinical signs, immediately after hibernation. The tortoises that presumptively became infected later on during the late spring/summer (nos. 10, 11, 12, and 13) had clinical signs that lasted for nearly one month before their death.

Of 16 TeHV3-infected tortoises that died during the overall period of observation, four (nos. 6, 7, 8, 18) were submitted for pathologic, bacteriological and virological investigations, whereas the others (nos. 1, 2, 3, 4, 5, 9, 10, 11, 12, 13, 15, and 17) were frozen and only virological examination was carried out. PCR confirmed all cases as TeHV infected and the virus was identified as a member of the TeHV3-A genogroup. Tortoise no. 18, asymptomatically infected, was a *T*. *marginata* that survived the acute stage of the outbreak: it died suddenly in the summer of 2014, but was negative for TeHV DNA by PCR at the time of death and for 15 days prior (see below).

Results of bacteriological isolation performed on the dead animals were unremarkable.

After the initial assessment of the ongoing infection due to TeHV3 during 2013, the semi-nested PCR protocol for TeHV3 was implemented as test of choice because of its higher sensitivity (6-fold more sensitive than the protocol of vanDevanter et al. [15]). PCR was also performed on all tortoises suspected to have been exposed and some of the non-exposed, which were randomly selected from those housed in adjacent enclosures in order to identify and separate other infected animals. Interestingly, all the tortoises that died had been housed in the same enclosure during the previous year (July 2012), together with the *T*. *marginata* (tortoise no. 18-see above), which was the only survivor of the group after the end of the initial phase of the outbreak. These observations were confirmed by 2012 camera recordings. Immediately after the onset of the outbreak (13 April 2013), the animals suspected of having been exposed to the infected individuals were separated, yet without a clear etiology of the outbreak. Moreover, biosecurity precautions and additional hygiene measures, including prompt identification and isolation of infected and ill animals, immediate separation of any suspected infected case and affected groups, as well as appropriate clothing and environmental disinfection, were implemented. Soil inside the enclosures was removed and the equipment was washed with a virucidal agent (Virkon S tablet, 1% solution, DuPont, Milan, Italy). Overall 22 tortoises were considered exposed based on the contact with confirmed cases and infected animals, which were all PCR positive for TeHV DNA, with the exception of two tortoises (nos. 21 and 22), that remained negative throughout the duration of the study. All the other non-exposed tortoises (those without contact of confirmed cases or infected animals) were sampled repeatedly (once a month at the beginning of the outbreak and then every two months except during the hibernation) and remained PCR negative. Based on PCR results, infected tortoises were isolated in different gardens. Tortoises housed in un-affected enclosures did not become clinical.

**Fig 1:**
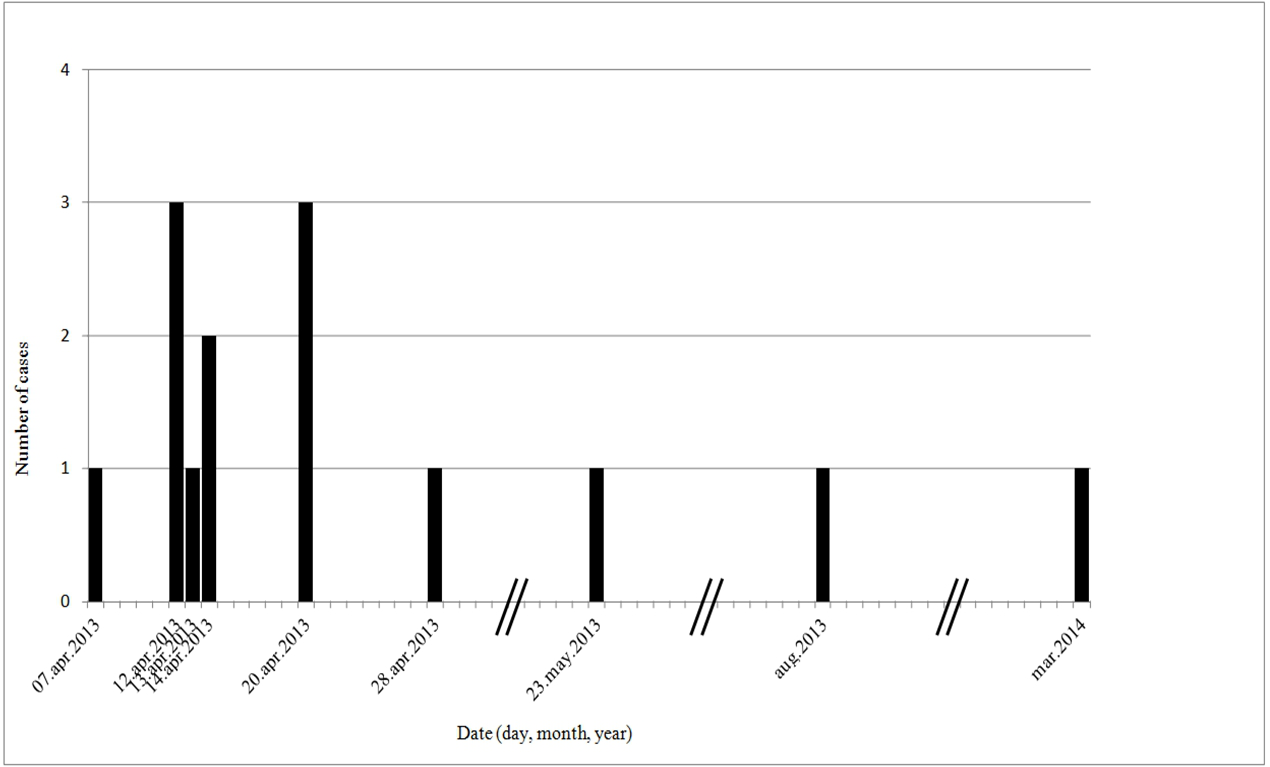
Epidemic curve of the TeHV3 outbreak, April 2013-March 2014. The curve was built using only the cases having clinical signs, for which it is possible to estimate an exact date of onset of the clinical signs. Bars represent longer periods of time that were cut in the figure. The pattern of the curve, with its irregular trend of cases reflecting the timing and extent of repeated exposures, is consistent with an intermittent source of infection. An estimation of the incubation period was attempted observing the gaps between the peaks of the curve that could represent tortoise-to-tortoise transmission events, followed by a possible incubation period. After the initial period, the outbreak waned, perhaps because of the successful and rapid implementation of control measures.

The pattern of the epidemic curve (Fig 1) indicates an intermittent source and the estimation of the incubation period ranged between 5 and 10 days. After the initial period, the outbreak waned.

With the exception of a suspected hatchling (see below), the outbreak affected only adults. The overall cumulative incidence (whole collection) was 7.4% (20/270), incidence rate 0.006 animal/month, morbidity 5.5% (15/270), mortality 5.5% (15/270). However, considering the group of exposed animals (n = 22) only, the cumulative incidence was 90.9% (20/22), incidence rate 0.44 animal/month, morbidity 68.2% (15/22), mortality and case-fatality 75% (15/20).

The semi-nested PCR protocol was also used to monitor long-term a group of asymptomatic PCR positive tortoises (nos. 14, 15, 16, 17, 18, 19, and 20). Repeated samplings were performed on this group of animals during spring/summer and pre-hibernation of the year 2013 and again at post-hibernation, spring/summer and pre-hibernation of the year 2014. Tortoises were either intermittently (no. 14, 16, 17, 18, 20) or permanently (no. 19) PCR positive (Table 1). Among the intermittent shedders, TeHV3 DNA was detected at different times of the year, but especially during post-hibernation: 66.6% of the asymptomatic infected tortoises were positive during post-hibernation *vs* the 25–50% of the positivity in the other periods (the percentages consist of different single positive tortoises in the different periods).

At the end of the observation period (December 2014), only two of the exposed and infected *T*. *graeca ibera* (nos. 14 and 19) and two *T*. *hermanni hermanni* (nos. 16 and 20) were still alive.

Several of the infected tortoises (nos. 13, 14, 15, 16, 17, and 20) were already positive at the time of the deposition and were able to lay eggs during the 2013. Of them, only no. 13 had clinical signs consisting of hind limb paresis at the time of the deposition. Regardless of the birthplace, incubator or ground, the newborn tortoises were kept altogether, but separated from all the other animals. Interestingly, mild signs of conjunctivitis were present at birth in one hatchling that was born in isolation in the incubator from an egg laid by the infected tortoise no. 20. This newborn tortoise died the following year, during post-hibernation. Overall other 8 hatchlings (3 born from the infected tortoises nos. 13, 14, and 16, and 5 born from non infected tortoises) of the same age died without any detectable clinical sign. The mortality rate was considered within the normal range for 1-year old tortoises. The tissues (liver, lung, and spleen) of four of these dead hatchlings, including that with signs of conjunctivitis, all born from eggs of infected tortoises (nos. 13, 14, 16 and 20), were submitted for TeHV3 PCR. Necropsy was not performed because the carcasses had been frozen. Specimens of these tortoises were processed separately from the others. Interestingly, only the tissues of the hatchling that had signs of conjunctivitis was positive by PCR detecting the partial sequence of the UL39 gene of the TeHV3, whereas the others were negative. The PCR product was sequenced to confirm the identity of the viral DNA (GenBank accession no. KU991750). In order to further confirm this result and exclude any possible contamination, a new DNA extraction was carried out from the tissues of the four young hatchlings that were stored at -80°C; the PCR targeting the partial sequence of the UL39 gene was repeated in another laboratory (University of Bern) and the previously obtained results were confirmed by PCR and sequencing consistently with a likely vertical transmission of the virus.

### Lesions

**Fig 2:**
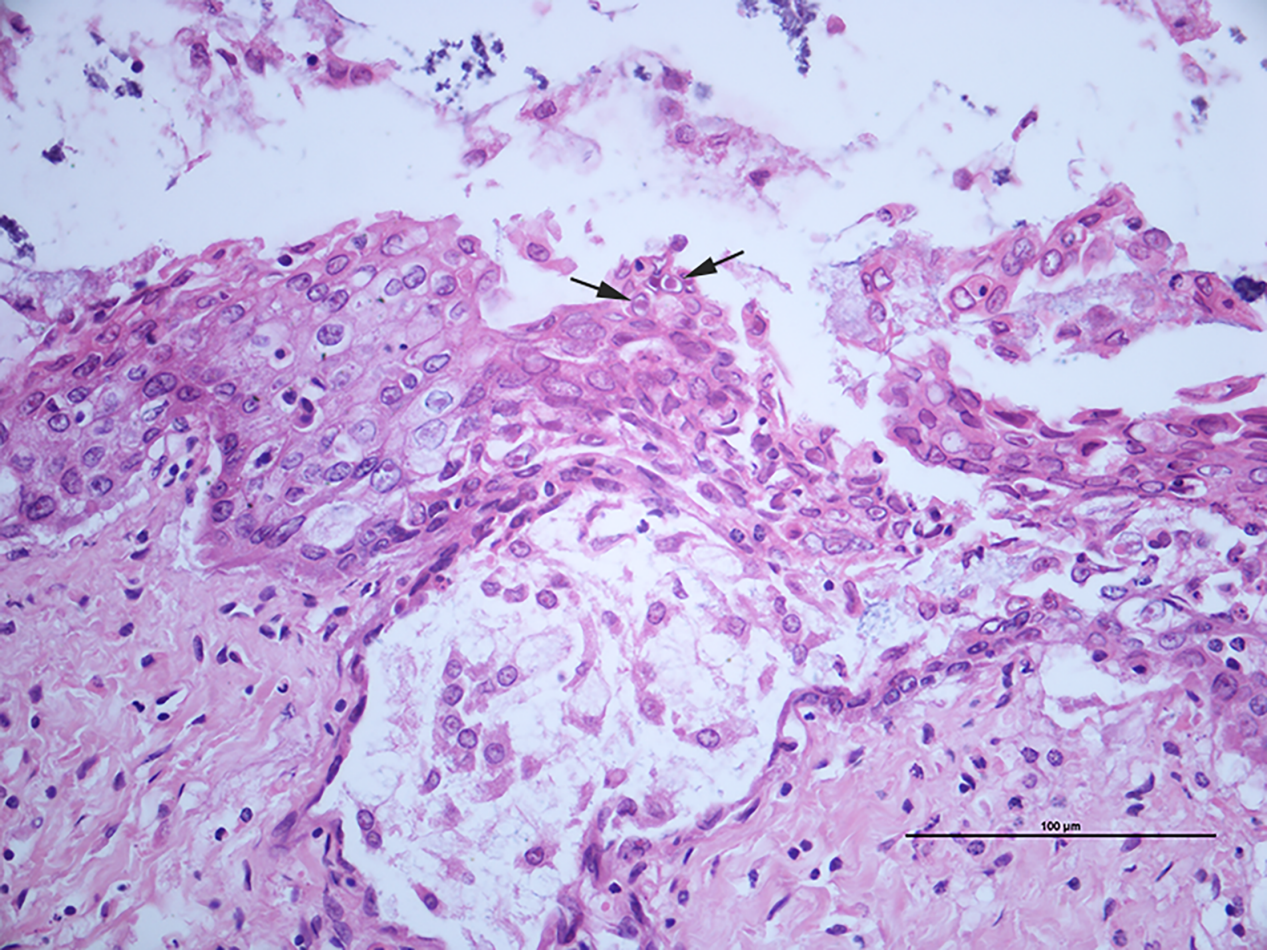
Tortoise no. 7: Oral cavity, glottis. Mucosal erosion and ulceration with marked spongiosis (intercellular oedema). Note the presence of intranuclear amphophilic inclusions in the epithelial cells (arrows) associated with margination of the nuclear chromatin. H&E, 400X.

The histological lesions of the three necropsied tortoises that died during the outbreak (tortoises nos. 6, 7 and 8, all *T*. *hermanni hermanni*) included: a) diffuse, moderate hyperplasia of the esophageal mucosa (tortoises nos. 6 and 7); b) sloughing of the epithelial cells and multifocal erosion (tortoise no. 6); c) ulceration and hyperplasia of the glottal epithelium associated with heterophilic pustules, epithelial necrosis and fibrin (tortoise no. 7); d) intranuclear amphophilic inclusion bodies (tortoises nos. 6 and 7, Fig 2). Tortoise no. 8 showed moderate diffuse heterophilic bronchitis and pneumonia, without detectable inclusion bodies.

Tortoise n. 18, the only survivor in enclosure n. 1, an intermittent shedder, died suddenly in August 2014, after one year from the peak of the outbreak and at this time was negative by PCR for TeHV3 DNA(OSW and tissues-liver, spleen, and lung). No significant gross lesions were observed at necropsy and histology revealed a moderate, multifocal heterophilic stomatitis associated with diffuse, mild bronchitis and pneumonia with no inclusion bodies.

### Sequencing

The sequences of the amplicons obtained by PCR targeting the helicase-primase complex gene of the tortoise no. 6, and those of the UL39 gene of the tortoises nos. 6, 18 (obtained from the specimens collected during both 2011 and 2013) and of the positive hatchling were submitted to GenBank and an accession number was assigned to each of them. The partial sequence of the helicase-primase complex gene [6] (GenBank accession n. KU991746) shared 98–100% identity with the homologous TeHV3 sequences reported in GenBank (accession nos. DQ343884, DQ343889, DQ343891, DQ343892, AY188757). The alignment of the partial sequences of the UL39 gene [11] amplified by PCR in our study (GenBank accession nos. KU991747, KU991748, KU991749, KU991750) indicated that they were identical (S2 Fig) and shared 98–100% identity with the homologous sequences of TeHV3 reported in GenBank (accession nos. AY338245, DQ343895, DQ343896, DQ343897, DQ343899, DQ343901). Overall, the results of the sequencing characterized the virus as TeHV3 genotype, in accordance to criteria previously reported [3] and further typed into genogroup A.

### ELISA

Serology was performed on samples collected from the exposed tortoises no. 18, 19, 20, 21, and 22, still alive at the time of the serological investigation. The nos. 18, 19, 20 were positive, whereas nos. 21 and 22 were negative. No. 18 had the highest OD reading (data not shown).

Serum samples from the other 24 non-exposed tortoises, housed in the enclosures adjacent to those of the infected tortoises were also submitted for serological examination. Fourteen samples were negative, 2 were positive, whereas 8 samples had doubtful results. The two serological positive non-exposed tortoises were an adult male that was born in another breeding facility, and a young male that had always been kept separate from the infected tortoise enclosures and was not in the collection at the time of the outbreak. These ten tortoises resulted either ELISA positive (n = 2) or doubtful (n = 8) were sampled repeatedly following the same schedule used for the asymptomatic infected and the other non-exposed animals and when tested by PCR they were always negative for the presence of TeHV3 DNA.

### Risk factors

Table 2 reports the most significant risk factors associated with the TeHV3 outbreak, obtained with the case-control study. However, species, age and gender resulted as confounding factors by Mantel-Haenzel method (data not shown). This was expected considering that the breeder was used to locate tortoises based on age and gender. Accordingly, the main significant factor remains the location of the tortoises in specific enclosures. Anyhow, the death of a *T*. *hermanni* with TeHV infection was 8.36 times more likely (OR = 8.36, CI 95%: 1,04–67,09, *P* = 0,03) than a *T*. non-*hermanni*, also considering the correction for the confounding factor.

**Table 2:** Attack rate and pooled Odds Ratio (OR), with corresponding 95% confidence interval (95% CI), of different risk factors for the tortoises exposed to TeHV3 infection.

Risk factor	cases and infected tortoises, *n*	not infected tortoises, *n*	total, *n*	Attack rate (%)	OR	95% CI	*p*-value
species							
*T*. non-*hermanni*^*1*^	3	21	24	12,5	1	-	-
*T*. *hermanni*	17	43	60	28,3	2,77	0,73–10,5	0,16
gender							
female^1^	8	19	27	29,6	1	-	-
male	12	9	21	57,1	3,17	0,96–10,57	0,05
not indentified#	0	36	36	0	na		na
age							
<10 years^1^	2	42	44	4,5	1	-	-
≥10 years	18	22	40	45	17,18	3,65–80,88	<0,001
location of the 2012							
other enclosures^1^	2	58	58	3,5	1	-	-
enclosure no.1*	10	0	11	90,9	156,4	6,92–2367	<0,001
enclosure no. 2†	8	7	15	53,3	20,67	3,89–109,6	<0,001
location of the 2013, initial phase							
other enclosures^1^	0	62	62	0	1	-	-
enclosure no.1	7	0	0	100	68,92	3,68–1291	<0,001
enclosure no.2	3	2	5	60	5,47	0,84–35,42	0,08
enclosure no.3	3	0	3	100	22,59	1,08–472,7	0,01
enclosure no.4	7	0	7	100	68,92	3,68–1291	<0,001
location of the 2013, second phase							
other enclosures^1^	0	62	62	0	1	-	-
enclosure no.1	1§	0	1	100	9,14	0,29–286,1	0,27
enclosure no.2	6§	2	8	75	20,67	3,6–118,5	<0,001
enclosure no.3	1§	0	1	100	9,14	0,29–286,1	0,27
enclosure no.4	7§	0	7	100	112	5,8–2165	<0,001

^1^ Reference category.

na: not applied.

# The gender of the tortoises is not identified until they are 6 years old.

* All tortoises in this enclosure were male.

† All tortoises in this enclosure were female.

§The other infected tortoises were dead.

### Retrospective investigation

The retrospective investigation carried out on 38 OSW DNA, collected during the 2011 post-hibernation period from the same adult tortoises affected by the outbreak, revealed that only *T*. *marginata* no. 18 was already PCR positive for TeHV3 prior to the outbreak. This tortoise was donated three years before the outbreak and had been quarantined for the first year, after which it was housed in an enclosure with young tortoises. It was born in captivity in a breeding facility with a previous history of herpesviral infection. However, no clinical signs had ever been observed previously in this animal, with the exception of a “wet” nose mentioned by the breeder that was considered a frequent occurrence in this species and did not raise a specific health concern. The breeder remembered that during the summer 2012, 15 days before identifying the definitive location of the suspected enclosure housing infected animals, this tortoise had been taken to a nursery school to be shown in a didactic activity on pet animals, according to procedures indicated by the CITES. Reactivation of the latent virus following this likely stressful event cannot be ruled out.

## Discussion

The current paper describes the molecular, serological, and epidemiological characterization of a TeHV3 outbreak that occurred in a large private captive tortoise collection aiming to partially unravel the dynamic of the infection. Molecular tests were performed to confirm the clinical diagnoses, identify clinically healthy infected animals and to characterize the infecting TeHV genotype and genogroup. Statistical analysis aiming to evaluate the presumptive role of a number of risk factors possibly influencing the occurrence and the course of the infection were also carried out. Furthermore, here we present the first evidence of the most likely vertical transmission of TeHV3.

PCR is a rapid test to detect viral shedding in infected carriers. Identification of infected animals allowed the rapid implementation of management recommendations including their prompt separation from those un-infected. PCR detects the presence of viral DNA, which is generally considered evidence of an ongoing infection. However, if the virus is latent and not shed at the time of sampling, the test would provide a negative result. Alternatively, serology allows the detection of the exposure of TeHV3 on the basis of the presence of anti-TeHV3 circulating antibodies, which arise starting 4 to 8 weeks after the onset of infection and that are still detectable in absence of viral shedding. Interestingly, the ELISA confirmed the absence of seroconversion of two exposed animals (nos. 21 and 22), which also remained PCR negative for the duration of the outbreak and the post-outbreak monitoring, consistent with the absence of infection in these individuals. All non-exposed tortoises with positive (n = 2) and questionable (n = 8) serological results in the present study were submitted for PCR to detect TeHV DNA, but they were consistently negative. The two seropositive non-exposed tortoises could have already seroconverted prior the outbreak without shedding virus during the current outbreak; alternatively, they may have been false positive [16]. The results stress that no single test is able to conclusively define the infectious state of an individual and consequently repeated sampling followed by serological, and molecular testing, together with pathological and clinical assessment, is always highly recommended.

Thanks to the retrospective investigation it was possible to identify *T*. *marginata* no. 18 as the “patient 0”, likely responsible for the introduction of TeHV3 into the breeding facility. It was the only survivor among the exposed tortoises in enclosure no. 1 and had the highest OD value at the ELISA. This individual originated from another collection, where it probably contracted the viral infection. It was quarantined for the first year after its arrival in the new collection and remained there for 3 years, prior to the outbreak. This finding is a prime example of the consequences of viral reactivation from latency and how even long quarantine periods may not protect from the entry of pathogens into reptile collections when not complemented with appropriate screening tests for relevant pathogens. The infection remained undetected for at least two years despite contact between infected and non-infected tortoises, together with a viral shedding retrospectively documented since 2011.

The epidemiological investigation was instrumental in highlighting possible risk factors and occurrences that favor TeHV3 spread. Camera recordings, initially used to monitor mating, became a very useful tool to monitor animals, the dynamic of the contagion and to confirm animal movement. All cases and infected tortoises were located in four enclosures, with three of them adjacent to each other and one separate. Interestingly, the first cases detected during the outbreak had been housed in the same fenced enclosure in the summer of 2012. However, no evidence of infection was observed in that year. Statistical analysis identified the location of specific enclosures as the most relevant risk factor. However, in the second phase of the outbreak (after 13 April 2013), this link was likely artificially magnified by the separation of the affected animals to isolate them from the clinically healthy tortoises. In any case, the findings are consistent with an unlikely transmission of the virus across enclosures. The infection reached non-adjacent enclosures probably via the translocation of animals operated by the breeder. Considering the whole population at risk (n = 270), the spread of the pathogen was likely limited because of the parceled arrangement of the collection in restricted groups and in low stocking density fenced enclosures, prior to the outbreak. In conclusion, the transmission of the virus in the present outbreak appears to have occurred secondary to direct contact with infected animals suggesting that long distance aerosol or other indirect manner of viral transmission are unlikely. Biosecurity procedures undertaken by the breeder might have also played a role in reducing the viral spread in the collection.

The variability of the estimated incubation period, based on epidemic curve and ranging between 5 and 10 days, might depend on a series of variables including the contacts with infected tortoises occurring at different times, varying amount of virus shed by the carrier tortoises resulting in variable viral loads that either met or did not meet the threshold required for successful infection (unknown) and changes in environmental temperature. However, the current study documents an outbreak that occurred in a captive environment where factors like density, which although low for captive conditions but in any case higher than in the natural conditions, could have favored the high morbidity and mortality observed [17].

The epidemiological investigation reveals that the majority of the cases with rapid and fatal outcome occurred during post-hibernation, whereas cases that occurred later had a longer clinical course. Interestingly enough, two animals that initially survived the outbreak (nos. 13 and 17) died the following year during the post-hibernation period. Post-hibernation was previously hypothesized to be a major risk of morbidity and mortality [5,17]. Our findings show that post-hibernation was also the time of the year when the highest number of viral shedders were detected, consistent with that predicted in the literature [5,17]. The reason for which the post-hibernation appears to be a critical time for the epidemiology of the disease is not completely understood. However, in this period the lymphoid organs of chelonians are depleted of lymphocytes. Furthermore, during the early spring, the highest peak of corticosterone is observed in the blood of these animals along with the highest peak of testosterone in males and the first peak of estrogen in females, that can play detrimental effect on the adaptive immune functions, known to be critical against viruses [18,19,20]. Finally, the environmental temperature during post- and pre-hibernation is closer to the optimal TeHV3 replication temperature than during the summer [5]. All these aspects might contribute to promote the replication of the virus in a host variably, temporary and physiologically immunosuppressed, partially explaining the epidemiological results observed in this study.

A possible effect of the environmental temperature on clinical cases was investigated in the current study but no statistical evidence was found. However, the power of the statistical analysis was limited because of the small sample size available (n = 12) and a larger sample size along with more extensive investigations are needed to draw more definitive conclusions on this aspect.

The infecting strain was determined to belong to the TeHV3 genotype, genogroup A. Genogroup A appears to be the most common circulating TeHV3 genogroup of the two, A and B, known to date [5]. Both genogroups A and B were found to be lethal. However, in tortoises infected with the genogroup B viral pneumonia, vasculitis and/or perivasculitis and disease of the central nervous system were overrappresented once compared to those developing in tortoises infected with strains of the genogroup A [5]. The virus involved in the present outbreak was responsible for a high case-fatality rate, similar to other reports of infection caused by the same viruses [9,21,22].

However, mortality was limited to a few exposed animals, including some of the oldest animals in the collection, with the highest sentimental value. The most affected species during the outbreak was *T*. *hermanni*, the most common species in the collection. However, three different species (*T*. *hermanni*, *T*. *graeca* and *T*. *marginata*), sharing the same enclosures, were infected. No statistical difference of species was found with respect to the infection, whereas the higher significant risk of mortality was observed in *T*. *hermanni* than in the group consisting of all *T*. non-*hermanni*, supporting the anecdotal reports describing Hermann’s tortoises being highly sensitive to the disease [3]. Consistently, all infected males of *T*. *hermanni* (n = 10) died, whereas all infected *T*. *graeca* (n = 2) survived. The only infected *T*. *marginata* died, but it was negative to TeHV3 PCR on OSW and tissues at the time of the death.

Neurological signs were previously reported in infected tortoises [2,3,10] and TeHV3 is known to be strongly neurotropic in tortoises [11], similar to herpesviruses infecting other animal species [23,24,25]. The neurological case that occurred in the present outbreak had a long clinical course, with a fatal outcome. Unfortunately, no histological examination was carried out and no unambiguous association between viral infection and putative lesion of the central nervous system could be determined; however, this is likely to have occurred similarly to what reported also in other species [2]. However, further studies are needed to clarify this aspect.

The results of testing the six infected but asymptomatic tortoises, carried out for two years, showed a wide range of patterns of viral shedding that was either intermittent or persistent, further highlighting its unpredictability [5].

Finally, in this article we provided the first evidence for the most likely vertical transmission of TeHV3. The hatchling born from the egg of the intermittent shedder no. 20, was placed in an incubator and had never been in contact with any infected animal. This tortoise hatched with signs of conjunctivitis, died the following year at post-hibernation, and was PCR positive for TeHV3 at the time of death. No clinical signs were observed in any of the other hatchlings housed together and none of them was positive by PCR consistently with having no role in infecting the positive hatchling, further supporting a vertical transmission of the virus. The clinical history, management and diagnostic results concerning this tortoise are consistent with a vertical transmission of the virus. A specific transmission study is necessary to further support this new proposed way of viral transmission conclusively clarifying this pivotal aspect of TeHV3 pathogenesis.

## Conclusion

Of the TeHV associated outbreaks described in the literature none has been analyzed to the extent of this study. The breeding facility affected by the outbreak had excellent management and biosafety measures, which however did not prevent the occurrence of the herpesvirus infection. Quarantine alone, even for extended periods, might not be sufficient to prevent a TeHV3 outbreak. Many of the procedures performed during the outbreak, including the separation of the animals into small groups, are likely to have reduced the spread of the infection. Laboratory testing had a critical role to formulate urgent management implementations. Evidence of direct and vertical transmission of TeHV3 along with a wide range of viral shedding in asymptomatically infected animals have been highlighted.

Many other issues still need to be clarified regarding TeHV3 infection, including the actual host susceptibility, the conclusive proof of vertical transmission and the existence of a seasonal trend or the complex interaction between host, environment and virus, especially in natural conditions.

## Supporting information

S1 Fig Map of the enclosures where chelonians were located. (TIF)

S2 Fig Alignment of the partial sequences of TeHV3 ribonucleotide reductase-large subunit, UL39 gene, obtained from tortoises nos. 6, 18 (including both the 2011 and 2013 samplings) and from the positive hatchling. The alignment obtained by MAFFT software shows full nucleotide identity among the compared sequences.
(TIF)

S1 Table PCR protocols used for herpesviruses detection in tortoises. (DOC)

S1 Video TeHV3 infected tortoise with neurological signs. This female of *Testudo hermanni*, over 30 years old, had neurological signs, consisting of hind limb paresis that lasted all year long. At the onset of the paresis, the tortoise was able to lay 3 eggs. It then partially recovered from the neurological syndrome, but died during post-hibernation of the following year, after the onset of oral discharge. This tortoise was always positive for TeHV DNA from OSW until its death.
(MP4)

S1 Appendix Investigation on the possible effects of the temperature during the TeHV3 outbreak and proposal for a statistical model. (DOCX)
