# Supplementary figures and images for: Clinical, virological and epidemiological characterization of an outbreak of Testudinid Herpesvirus 3 in a chelonian captive breeding facility: Lessons learned and first evidence of TeHV3 vertical transmission
Source: PLoS One. 2018 May 10;13(5):e0197169. doi: 10.1371/journal.pone.0197169 (PMC5944942; doi:10.1371/journal.pone.0197169)

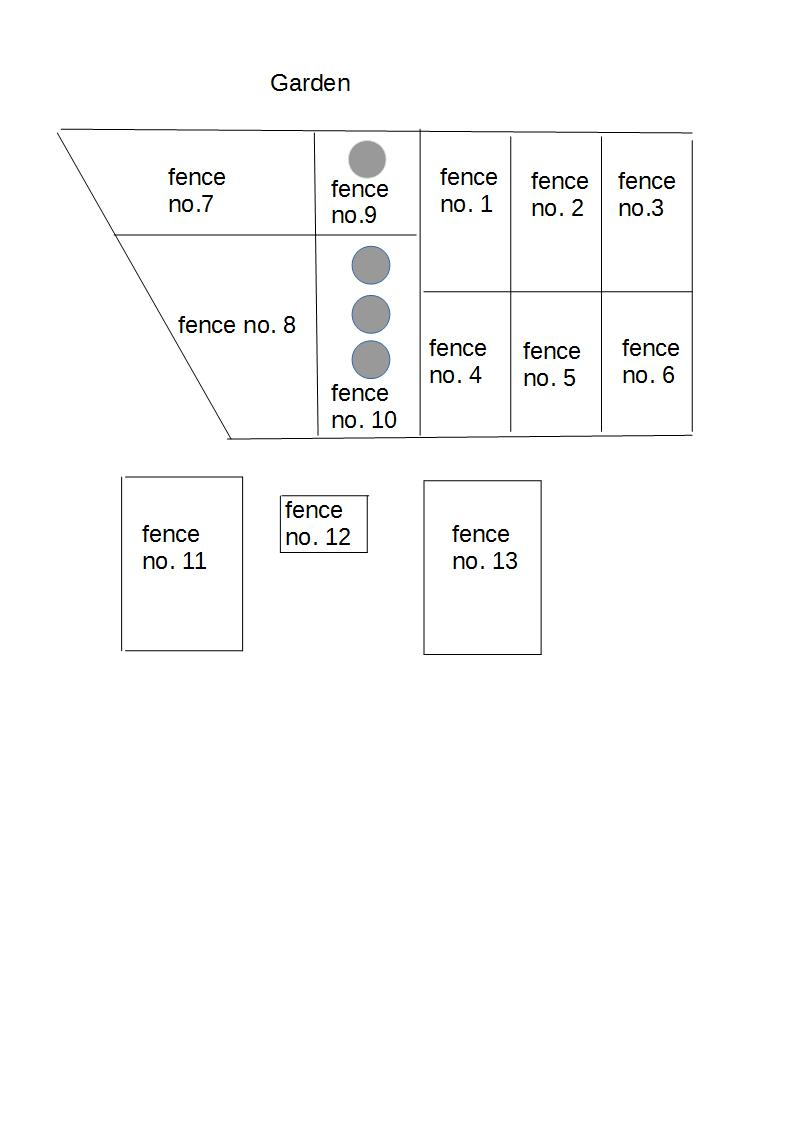

Supplement: S1 Fig — (TIF) [file pone.0197169.s001.tif]

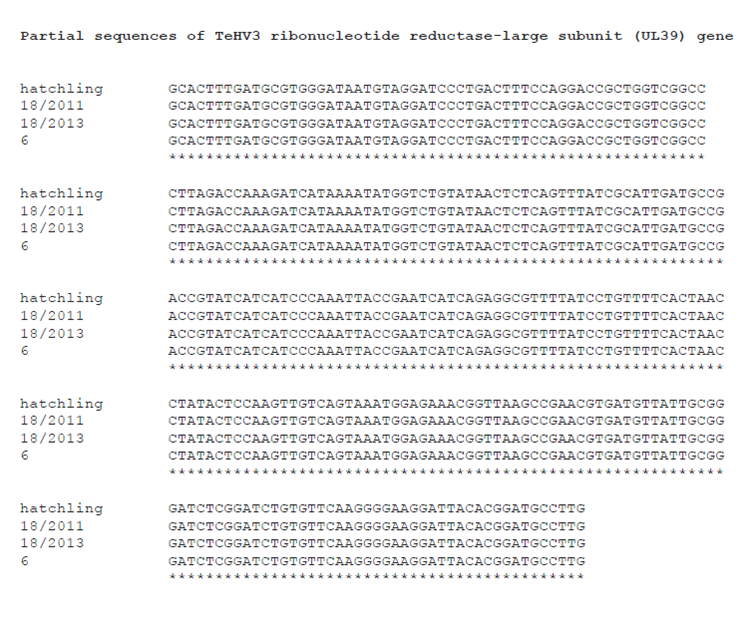

Supplement: S2 Fig — The alignment obtained by MAFFT software shows full nucleotide identity among the compared sequences. (TIF) [file pone.0197169.s002.tif]
