# Supplementary material for: Clinical, virological and epidemiological characterization of an outbreak of Testudinid Herpesvirus 3 in a chelonian captive breeding facility: Lessons learned and first evidence of TeHV3 vertical transmission
Source: PLoS One. 2018 May 10;13(5):e0197169. doi: 10.1371/journal.pone.0197169 (PMC5944942; doi:10.1371/journal.pone.0197169)
Supplement: S1 Table — (DOC) [file pone.0197169.s003.doc]

**S1 Table. PCR protocols used for the detection of herpesviruses in tortoises.**

| PCR assay | Target gene | Primer sequence (5'-3') | Amplification profile | | | | | Product size (bp) | PCR type | **References** |  |
| --- | --- | --- | --- | --- | --- | --- | --- | --- | --- | --- | --- |
|  |  |  | Initial denaturation | denaturation | annealing* | extension | final extension |  |  |  | |
| Consensus panherpesvirus | DNA polymerase | DFA (F):  GAYTTYGCNAGYYTNTAYCC |  | 40 cycles | | |  |  |  |  | |
|  |  | ILK (F): TCCTGGACAAGCAGCARNYSGCNMTNAA | 94 °C,  2 min | 94 °C,  30 sec | 46 °C,  1 min | 72 °C,  1 min | 72 °C,  5 min | 725 (DFA+KG1)  470 (ILK+KG1) | conventional | VanDevanter et al. (1996) | |
|  |  | KG1 (R): GTCTTGCTCACCAGNTCNACNCCYTT |  |  |  |  |  |  |  |  | |
|  |  | TGV (FN): TGTAACTCGGTGTAYGGNTTYACNGGNGT |  | 45 cycles | | |  |  | nested |  | |
|  |  | IYG (RN): CACAGAGTCCGTRTCNCCRTADAT | 94 °C,  2 min | 94 °C,  30 sec | 46 °C,  1 min | 72 °C,  1 min | 72 °C,  5 min | 225 |  |  | |
| TeHV-3 | helicase-primase complex (UL5) | F2 (F):  TGGTTGGACAACAACATGGC |  | 40 cycles | | |  | 307 | conventional | Teifke et al. (2000) | |
|  |  | R2 (R):  TCATTCCGTTATACATGAGCG | 94 °C,  5 min | 94 °C,  30 sec | 60 °C,  30 sec | 72 °C,  45 sec | 72 °C,  5 min |  |  |  | |
| TeHV-3 | ribonucleotide reductase-large subunit (UL39) | OS (F):  TGCACTTTGATGCGTGGGAT |  | 35 cycles | | |  | 386 | conventional | Origgi et al. (2004) | |
|  |  | OAS (R):  TTGATCGTATTCGAATGCCG | 94 °C,  5 min | 94 °C,  30 sec | 58 °C,  30 sec | 72 °C,  45 sec | 72 °C,  5 min |  |  |  | |
|  |  | RN:  CAAGGCATCCGTGTAATCCT | 94 °C,  5 min | 94 °C,  30 sec | 60 °C,  30 sec | 72 °C,  45 sec | 72 °C,  5 min | 284 | semi-nested | the present study | |

F= forward primer of the first round of PCR; R= reverse primer of the first round of PCR; FN= forward primer of the second round of PCR;

RN=reverse primer of the second round of PCR.

* the annealing temperatures were chosen through gradient PCRs, with the exception of the protocol of VanDevanter et al., 1996, for which the annealing temperature indicated in the original paper was used.
