# Supplementary material for: Clinical, virological and epidemiological characterization of an outbreak of Testudinid Herpesvirus 3 in a chelonian captive breeding facility: Lessons learned and first evidence of TeHV3 vertical transmission
Source: PLoS One. 2018 May 10;13(5):e0197169. doi: 10.1371/journal.pone.0197169 (PMC5944942; doi:10.1371/journal.pone.0197169)
Supplement: S1 Appendix — (DOCX) [file pone.0197169.s005.docx]

**Investigation on the possible effect of the temperature on the TeHV3 outbreak.**

Although a significant body of literature has been written on TeHVs, the dynamic of the host-pathogen-interaction is not fully understood, along with potential role of the environment. Reptiles are poikilothermic, and temperature becomes a variable that could further influence the occurrence and the evolution of the disease. The effect of the temperature on clinical severity, mortality, and incubation has been partially investigated previously in other viral infections of vertebrate poikilotherms [1-6]. However, limited information is available concerning these aspects on tortoises infected with TeHVs.

A time-stratified case-crossover study was carried out in the context of the current outbreak of TeHV3 to assess possible effects of the environmental temperature on the infection.

**Materials and methods**

**Study design and statistical analysis**

The relationship between temperature and TeHV3 infection was investigated using a time-stratified case-crossover design. In this study design each case served as control of itself, comparing the days of the same month and year in which the same tortoise was either ill or healthy, respectively [7,8,9]. The definition of case in this analysis is that used for the confirmed cases described in the materials and methods of main text (tortoises showing clinical signs and positive for the presence of TeHV DNA by PCR). This choice was based on the presence of the clinical signs in the affected tortoises, which was used as “marker” of the onset of the disease, differently from those asymptomatically infected that showed no signs and for which no onset of the disease could be determined. This study design is useful to control potential confounding factors, performing a perfect matching on all characteristics of the investigated subjects [10]. Conditional logistic regression model was used to calculate OR and 95% confidence intervals (CI) at lag 3, 4, 5 and 6 days before the event, estimated as possible incubation period [11]. Meteorological data on temperature (daily minimum, maximum, mean) and humidity (daily mean), corresponding to the area where the breeding facility was located, were obtained from the website [www.ilmeteo.it](http://www.ilmeteo.it). The main parameters of the weather when the peak of the outbreak occurred (April 2013 for clinical signs and probably the middle of March for the infection) are reported in Table 1. Considering that tortoises living in temperate climates might become active at 10-12°C, but that their metabolism reaches the best functional parameters at their preferred body temperature (around 28-30°C) [12,13], an environmental temperature of 10°C was assumed as non-optimal for tortoises. For these reasons, the effect of a mean daily temperature ranging 8–12°C (tr8-12) or 9–11°C (tr9-11) and a difference of daily maximum and minimum temperature higher than 10 degrees (dt-10) were investigated. *P-*values ≤0.05 was considered statistically significant. The analyses were performed using Stata software 11.2 (StataCorp, College Station, TX, USA).

**Table 1. Meteorological data for the months of March and April 2013.**

| MONTHLY STATISTICS | **March 2013** | **April 2013** |
| --- | --- | --- |
| **Mean temperature*** | 9,71 | 15,43 |
| **Minimum mean temperature*** | 4,97 | 8,40 |
| **Maximum mean temperature*** | 13,48 | 20,50 |
| **Mean humidity (%)** | 75,97 | 67,13 |
| **Rainy days** | 15/31 | 9/30 |
| * Celsius degrees (°C) |  |  |

**Results**

The confirmed cases included in the analysis were 12. No significant impact of the environmental temperature on the occurrence of TeHV infection was determined (Table 2). However, considering the small sample size, attention should also be paid to *p-*values approximately <0.1, obtained with temperature range 9-11°C, recorded 5 days before the onset of the clinical cases.

**Table 2. Odds ratio (OR) and corresponding 95% confidence intervals (95% CI) referring to the onset of the clinical signs based to the variations of the temperature.** Three temperature intervals were determined: (I) on a day with a range of temperature between 8 and 12 degrees (tr8-12); (II) a day with a range of temperature between 9 and 11 degrees (tr9-11); (III) and a day with a temperature difference (max – min) greater than 10 degrees (dt-10). The values obtained were compared with those determined for a day with an average temperature outside the temperature range (8 and 12 degrees), which was calculated at different days before the event (lag 3-6 days).

| **Temperature-dependent variable** | **OR** | **95% CI** | ***p*-value** |
| --- | --- | --- | --- |
| **tr8-12** |  |  |  |
| **lag 3** | 0,48 | 0,09 - 2,39 | 0,367 |
| **lag 4** | 1,22 | 0,36 - 4,17 | 0,749 |
| **lag 5** | 1,49 | 0,45 - 4,94 | 0,511 |
| **lag 6** | 2,71 | 0,61 - 12,04 | 0,19 |
| **tr9-11** |  |  |  |
| **lag 3** | 0,8 | 0,16 - 3,98 | 0,787 |
| **lag 4** | 1,68 | 0,49 - 5,69 | 0,408 |
| **lag 5** | 2,69 | 0,87 - 8,32 | 0,085 |
| **lag 6** | 2,72 | 0,73 - 10,18 | 0,136 |
| **dt-10** |  |  |  |
| **lag 3** | 3,28 | 0,41 - 26,51 | 0,266 |
| **lag 4** | 2,31 | 0,46 - 11,61 | 0,311 |
| **lag 5** | 1,27 | 0,40 - 4,03 | 0,683 |
| **lag 6** | 2,31 | 0,71 - 7,52 | 0,165 |

**Discussion**

The investigation on the effect of the temperature in the current TeHV3 outbreak was performed because we were interested in assessing the existence of a link between environmental conditions (temperature) and dynamic of TeHV-associated disease. No significant findings were found. However, the small sample size was most likely a limiting factor that reduced the power of analysis. Moreover, in addition to an interesting *p=*0,085 obtained with temperature range 9-11°C recorded 5 days before the onset of the clinical cases, the trend of the *p*-values for the group of the 6 days was nearer to significant results in all the three different temperatures analyzed. A lag time higher than 6 days also should be further investigated and this hypothesis is supported also by the results of the epidemic curve that found a range of 5-10 days as incubation period.

Further studies including a larger number of observations are needed to better understand the relationship between TeHV-associated disease and environmental conditions. The statistical analysis used in this study could represent an initial model to implement and to build upon using further variables.

**References**

1. Avunje S, Kim WS, Oh MJ, Choi I, Jung SJ. Temperature-dependent viral replication and antiviral apoptotic response in viral haemorrhagic septicaemia virus (VHSV)-infected olive flounder (*Paralichthys olivaceus*). Fish Shellfish Immunol. 2012;32(6):1162-70.
2. Allender MC, Mitchell MA, Torres T, Sekowska J, Driskell EA. Pathogenicity of frog virus 3-like virus in red-eared slider turtles (*Trachemys scripta elegans*) at two environmental temperatures. J Comp Pathol. 2013;149(2-3):356-67.
3. Bayley AE, Hill BJ, Feist SW. Susceptibility of the European common frog *Rana temporaria* to a panel of ranavirus isolates from fish and amphibian hosts. Dis Aquat Organ. 2013;103(3):171-83.
4. Vike S, Oelckers K, Duesund H, Erga SR, Gonzalez J, Hamre B, et al. Infectious salmon anemia (ISA) virus: infectivity in seawater under different physical conditions. J Aquat Anim Health. 2014;26(1):33-42.
5. Toffan A, Panzarin V, Toson M, Cecchettin K, Pascoli F. Water temperature affects pathogenicity of different betanodavirus genotypes in experimentally challenged Dicentrarchus labrax. Dis Aquat Organ. 2016;119(3):231-8.
6. Sim RR, Allender MC, Crawford LK, Wack AN, Murphy KJ, Mankowski JL, et al. Ranavirus epizootic in captive eastern box turtles (*Terrapene carolina carolina*) with concurrent herpesvirus and mycoplasma infection: management and monitoring. J Zoo Wildl Med. 2016;47(1):256-70.
7. Maclure M, Mittleman MA. Should we use a case-crossover design? Annu Rev Public Health. 2000;21:193-221.
8. Crescio MI, Forastiere F, Maurella C, Ingravalle F, Ru G. Heat-related mortality in dairy cattle: A case crossover study. Prev Vet Med. 2010; 97: 191-7.
9. Wilson LA, Morgan GG, Hanigan IC, Johnston FH, Abu-Rayya H, Broome R, et al. The impact of heat on mortality and morbidity in the Greater Metropolitan Sydney Region: a case crossover analysis. Environ Health. 2013; 12 (1):1.
10. Janes H, Sheppard L, Lumley T. Case-crossover analyses of air pollution exposure data: referent selection strategies and their implications for bias. Epidemiology. 2005; 16: 717-26.
11. Origgi FC, Romero CH, Bloom DC, Klein PA, Gaskin JM, Tucker SJ, et al. Experimental transmission of a herpesvirus in Greek tortoises (*Testudo graeca*). Vet Pathol. 2004; 41: 50-61.
12. Huot-Daubremont C. Étude de la thermoregulation de la tortue d’Hermann (*Testudo hermanni hermanni*) au cours de son cycle annuel, à l’aide d’une sonde intracorporelle – rythme nycthéméral et échelle termobiologique. Chelonii. 2002; 3: 145–54.
13. Mazzotti S, Pisapia A, Fasola M. Activity and home range of *Testudo hermanni* in Northern Italy. Amphibia Reptilia. 2002;23(3):305-12.
